# A major role for retrieval and/or comparison in the set-size effects of change detection

**DOI:** 10.1167/jov.21.13.2

**Published:** 2021-12-01

**Authors:** James C. Moreland, John Palmer, Geoffrey M. Boynton

**Affiliations:** 1 Department of Psychology, University of Washington, Seattle, Washington, USA

**Keywords:** divided attention, visual memory, change detection, set-size effects

## Abstract

Set-size effects in change detection have been attributed to capacity limits in a variety of processes, including perception, memory encoding, memory storage, memory retrieval, comparison, and decision. In this study, we investigated the locus of the effect of increasing set size from 1 to 2. The task was to detect a 90 degree change in the orientation of 1 or 2 briefly presented Gabor patterns in noise. To measure purely attentional effects and not another phenomena, such as crowding, a precue was used to manipulate relevant set size while keeping the display constant. The locus of the capacity limit was determined by varying when observers were cued to a single relevant stimulus. To begin, we measured the baseline set-size effect for change detection. Next, a dual-task procedure and a 100% valid postcue was added to test for an effect of decision: This modification did not reliably change the set-size effects. In the critical experiments, a 100% valid cue was provided during the retention interval between displays, or only one stimulus was presented in the second display (local recognition). For both of these conditions, there was only a relatively small set-size effect. These results are consistent with the bulk of capacity limits being in memory retrieval or comparison and not in perception, memory encoding, or memory storage.

## Introduction

A procedure frequently used to study the effects of divided attention is the manipulation of set size in the change detection paradigm. Change detection typically consists of successively presenting two displays of multiple stimuli and asking an observer to judge whether there has been a change between the displays in one or more of the stimuli (e.g. Griffin & Nobre, 2003; Keshvari, van den Berg, & Ma, 2013; Scott-Brown & Orbach, 1998; Woodman, Vogel, & Luck, 2012). Usually, the displays are shown with a brief separation to prevent transients from signaling the change. For success in this task, the observer must process the sensory input from the first display, encode and store the stimuli in memory, process the second display, retrieve information about the first display, compare the corresponding stimulus representations, and make a decision on the basis of these comparisons.

When displays contain multiple stimuli, any decline in performance compared to a single stimulus is called a set-size effect. Such effects have been attributed to a variety of processing stages, including perception (Pestilli, Carrasco, Heeger, & Gardner, 2011), memory (e.g. Awh, Barton, & Vogel, 2007; Luck & Vogel, 1997; Rouder, Morey, Cowan, Zwilling, Morey & Pratte, 2008; Wilken & Ma, 2004; Zhang & Luck, 2008), and decision (Scott-Brown, Baker, & Orbach, 2000). We pursue the question of which of these processing stages causes set-size effects in change detection.

## Alternative theories of change detection

To set the stage, we provide a brief review of theories that incorporate capacity limits in perception, memory, or judgment and decision. In perception, there are many theories that posit limited capacity and that predict set-size effects (reviewed in Pashler, 1998; Scharff, Palmer, & Moore, 2011b). At one extreme are theories suggesting a serial process (or “bottleneck”) that allows only one stimulus to be identified at a time (Broadbent, 1958; Lachter, Foster, & Ruthruff, 2004). Other theories are less severe, suggesting some kind of limited capacity or resource that is divided among relevant stimuli (e.g. Kahneman, 1973; Navon & Gopher, 1979). These theories predict that performance in divided attention tasks is impaired because the stimuli are in competition for the limited capacity in perceptual stages. Finally, there are theories that assume no capacity limits in the perception of simple features or feature contrast (e.g. Palmer, 1994; Scharff et al., 2011b).

Another way that perception can cause a set-size effect stems from sensory interactions that are not attentional. One of the best known such phenomena is crowding (e.g. Bouma, 1970; Pelli, Palomares, & Majaj, 2004). Crowding is a stimulus interaction that limits perception in a variety of tasks (reviewed in Whitney & Levi, 2011). Unless care is taken, increasing set size decreases the spacing between stimuli, thereby increasing crowding. These effects occur even with relatively small set sizes. In Busey and Palmer (2008), we found evidence of crowding in a search experiment with set size 8 and, in an unpublished follow-up study, some evidence of crowding with set size 4. Such crowding effects have been shown in memory as well as perceptual experiments (Tamber-Rosenau, Fintzi, & Marois, 2015). Another phenomenon that can be confounded with set size is stimulus heterogeneity. Increasing heterogeneity amplifies set-size effects in both visual search (Rosenholtz, 2001) and visual memory (Lin & Luck, 2009). Mazyar, van den Berg, and Ma (2012) have argued that heterogeneity is a major source of set-size effects. Finally, one can change the stimulus configuration in terms of the relation between neighboring stimuli. For example, local differences can make targets more visible and reduce set-size effects in search experiments (Nothdurft, 1993) or removing neighbors can reduce context cues and increase set-size effects in visual memory experiments (Silvis & Shapiro, 2014).

Theories of memory also describe a variety of capacity limits that can result in set-size effects (see reviews in Brady, Konkle, & Alvarez, 2011; Oberauer, Farrell, Jarrold, & Lewandowsky, 2016). The first display provides a set of study stimuli to be encoded, stored, and later retrieved. The second display provides a set of test stimuli to be compared to the corresponding study stimuli. Each stage of memory processing has the potential of imposing a capacity limit. For encoding, stimuli might be moved into memory serially (Becker, Miller, & Liu, 2013) or in parallel with capacity limits (Rideaux, Apthorp, & Edwards, 2015; Rideaux & Edwards, 2016). Storage limits might exist in terms of the number (Luck & Vogel, 1997) or of the quality (e.g. Keshvari et al., 2013) of the representations. For retrieval, some theories propose serial (McElree & Dosher, 1993) or parallel (McElree & Dosher, 1989) access, or perhaps an effect of interference during retrieval that depends on the number of relevant stimuli (Oberauer & Lin, 2016). A yet longer list of possibilities is described in the General Discussion. Each of these broad hypotheses—limited capacity in encoding, storage, or retrieval—can predict a set-size effect in the change detection task.

Following memory, the final stages of processing are judgment and decision and they too can cause set-size effects. At this point in processing, observers have the relevant information in memory and need to compare this information to make a response. We divide the judgment and decision processes into two parts. First, there might be a capacity limit in the comparison process between representations of the two displays (Angelone, Levin, & Simons, 2003; Farell, 1985; Fernandez-Duque & Thornton, 2000; Hyun, Woodman, Vogel, Hollingworth, & Luck, 2009; Mitroff, Simons, & Levin, 2004; Simons, Chabris, Schnur, & Levin, 2002). Second, there might be a capacity limit due to noise compounded across the multiple comparisons needed to make the final decision (Palmer, Verghese, & Pavel, 2000; Sperling & Dosher, 1986; Tanner, 1961).

### The present study

Our overarching goal is to find the primary locus of set-size effects. This goal follows from our prior work to understand divided attention in a variety of tasks. We also have three supporting subgoals that guide our investigation. In typical set-size experiments, set sizes are varied over a range of 1, 2, 4, and more stimuli. In contrast, our first subgoal is to measure the effect of increasing set size from just 1 to 2 stimuli because these effects reveal the initial source of capacity limits as the number of stimuli is increased from a single stimulus (e.g. Bae & Flombaum, 2013; White, Palmer, & Boynton, 2018; Williams, Hong, Kang, Carlisle, & Woodman, 2013). Effects of larger set sizes are expected to share these initial capacity limits as well as possibly adding capacity limits imposed by other processes. That is not to say that the largest set-size effect is between 1 and 2 stimuli. That depends on the overall difficulty of the task and for relatively easy tasks, performance for small set sizes is often at ceiling.

Our second subgoal is to measure set-size effects that are purely attentional and not due to non-attentional phenomena such as crowding (Tamber-Rosenau et al., 2015), stimulus heterogeneity (Lin & Luck, 2009), or other configural phenomena between stimuli (Silvis & Shapiro, 2014). By attentional effects, we narrowly focus on cases in which the phenomena are subject to top-down control. In other words, the effects can be driven by instruction or endogenous cues. To do this, rather than manipulating display set size, the number of relevant stimuli were manipulated using a 100% valid precue (e.g. Makovski, Sussman, & Jiang, 2008; Wright & Green, 2000). Manipulating relevant set size allows the visual displays to be identical in all conditions and thus holds constant any stimulus-driven effect (Palmer, 1994). This manipulation of relevant set size gives the best chance of measuring purely attentional effects.

Our third subgoal is to conduct an experiment relevant to research in both perception and memory. Change detection in memory research typically differs from its use in perception research by the choice of stimuli. Rather than using hard-to-discriminate stimuli, such as low-contrast Gabors or small luminance changes, memory research typically uses easy-to-discriminate stimuli, such as high-contrast bars, colored patches, or nameable objects (e.g. Hollingworth, 2003; Luck & Vogel, 1997; Wilken & Ma, 2004). To address both bodies of research, we used both hard-to-discriminate and easy-to-discriminable stimuli by varying stimulus contrast. To preface one aspect of our results, similar effects were found for both hard- and easy-to-discriminate stimuli. Thus, for our measurements, these two experimental traditions gave a common result.

### Overview of experiments

**Figure 1.**
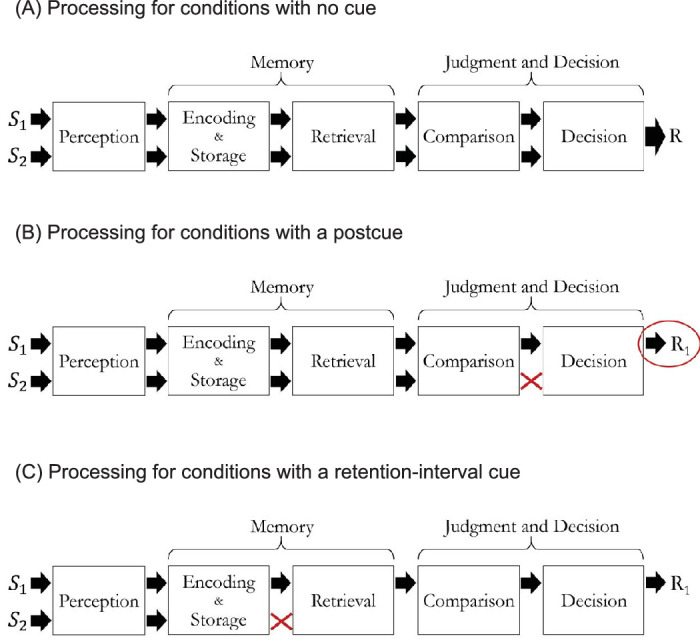
Schematic of the stages of processing necessary for three versions of the change detection task. Each stage is associated with one or more potential capacity limits. Panel (**A**) illustrates the basic change detection task of detecting a change anywhere in the display. Panel (**B**) illustrates the task used in Experiment 2 which was modified by a 100% valid postcue and instructions to detect a change specifically at the postcued location (e.g. R_1_). As marked by the red X, the postcue eliminated the need for decision processing of the uncued stimulus. Panel (**C**) illustrates the tasks used in Experiment 3 (and 4) which was modified by a 100% valid retention-interval cue (or local recognition). As marked by the red X, this cue eliminated the need for retrieval, comparison, or decision processing of the uncued stimulus.

In Experiment 1, we used a basic form of change detection in which two oriented Gabor stimuli were briefly presented in the first display, followed by a blank, and then a second pair of briefly presented Gabor stimuli. The observer's task was to report whether either Gabor changed in orientation from the first to the second display. Figure 1A shows a schematic of the processing stages that are associated with hypotheses for capacity limits in the basic change detection task: detect a change in any stimulus. The arrows show information about two stimuli moving through each stage. For this experiment, the set-size effect can arise anywhere in the processing sequence.

In Experiment 2, we eliminated the final decision as a possible source of the set-size effect. For the basic task of Experiment 1, the decision had a many-to-one mapping because a change in either side—in either Gabor—mapped to the same response. This means that any noise in the stimulus representations is combined for set size 2 but not for set size 1. To remove this contribution, a 100% valid postcue and independence across sides was introduced to make the stimulus response mapping one-to-one. The postcue was presented well after the second display so it does not aid in retrieval or comparisons between the displays. The changes are illustrated in Figure 1B: the postcue directs the decision process to a single stimulus (R_1_). In summary, Experiment 2 is intended to prevent the final decision process from contributing to the set-size effect.

In Experiment 3, we eliminated retrieval and comparison processes as possible sources of the set-size effect. This was done by adding a 100% valid retention-interval cue (often called a retro-cue) as illustrated in Figure 1C (reviewed in Souza & Oberauer, 2016). If a process such as memory encoding or memory storage limits performance, then the set-size effect with this modified procedure should remain the same as in the first two experiments because nothing about the displays or stimuli has changed up to this point. If, however, change detection is limited by memory maintenance, retrieval, or comparison, the set-size effect should be reduced or, in the extreme, eliminated. In Experiment 4, the results were further extended using a local recognition task (also called single-probe recognition) to distinguish memory maintenance from retrieval and comparison. In summary, these experiments allow progressive narrowing of the possible loci of capacity limits in change detection.

We conducted all of the experiments in two ways in separate sessions. In both, the judgment was of a coarse orientation change (90 degrees). First, low-contrast Gabor patches in noise were used as hard-to-discriminate stimuli, as is common in perceptual experiments. The contrast was chosen so that the mean performance with a single relevant stimulus was about 80% correct (50% is chance). Second, high-contrast Gabor patches were used as easy-to-discriminate stimuli, as is common in memory experiments. Under these high-contrast conditions, mean performance with a single relevant stimulus was about 98% correct. Using both conditions, we can make contact with both the perceptual and memory literatures.

## General method

### Observers

Twelve observers participated in each of the experiments. All observers had normal or corrected-to-normal vision. One of the observers was author J.M. All observers (except J.M.) were compensated $20/hour. All observers gave written and informed consent in accord with the human observers Institutional Review Board at the University of Washington, in adherence with the Declaration of Helsinki.

We determined the minimum number of observers needed to detect a set-size effect by conducting a power analysis based on pilot data from a previous Gabor detection experiment. In the previous experiment, observers (*N* = 5) each completed 1920 trials in a simple Gabor detection experiment comparing detection at one versus two possible cued locations (relevant set sizes 1 vs. 2). Other than using simple detection rather than change detection, the stimuli and procedure were similar to the present experiments. The observed set-size effect was 4.2% ± 1.1%. The standard deviation of this effect across observers was 2.4%. A power analysis was done for a yet smaller set-size effect of 2%. Using a paired sample, one-tailed *t*-test and a power of 80%, the minimum number of observers required was 11. For good measure, we chose to use 12 observers in each experiment.

### Stimuli and procedure

**Figure 2.**
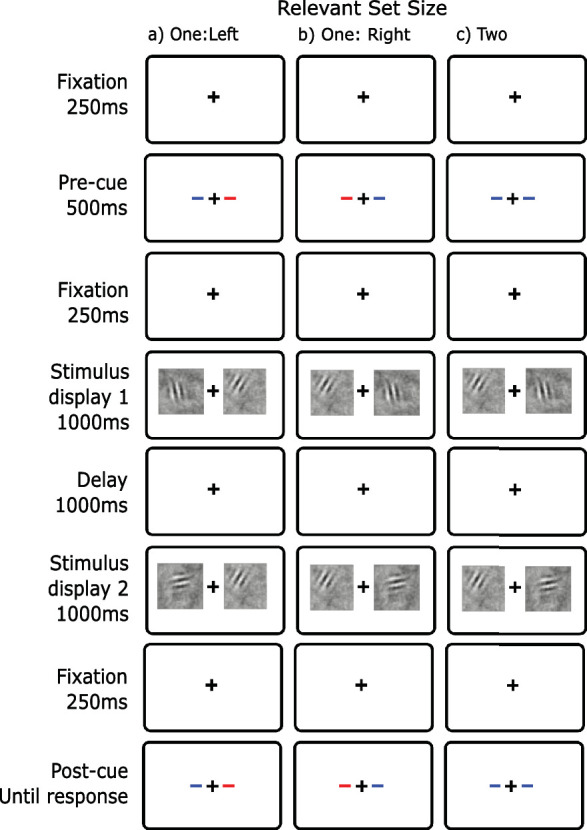
Display conditions for basic change detection (Experiment 1) with blue as the cue color. (**A**) relevant set size 1 - left; (**B**) relevant set size 1 – right; (**C**) relevant set size 2. Unlike this illustration, in all experiments, the entire screen was a middle gray. While the dynamic noise was displayed for 1000 ms, the Gabors were briefly displayed in a temporal Gaussian envelope with a standard deviation of 50 ms.

In all four experiments, the basic task was to detect whether the orientation of a Gabor in noise changed from a first display to a second display. Figure 2 shows a schematic of the procedure for each of the conditions of Experiment 1. As in our prior dual-task experiments (e.g. White et al., 2018), trials were blocked by condition: relevant set size 1 (left and right), and relevant set size 2. We blocked to make the task as simple as possible and thus maximize performance. For all conditions, observers began by foveating a fixation cross at the center of a gray screen (500 ms; 50% of max luminance). This was followed by a 100% valid precue consisting of two lines on either side of the fixation cross (1 degree eccentricity; 500 ms). For relevant set size 1, the lines were different colors (red and blue); for relevant set size 2, the lines were the same color. Each observer was allocated a cue color that indicated the relevant side (colors were counterbalanced across observers). An earlier version of the experiments did not have a precue with set size 2. However, there was no difference in the results for observers who ran under these conditions so data was collapsed for analysis.

Following the precue, a display containing two patches (6 degrees × 6 degrees) of dynamic noise appeared on either side of fixation. They were centered at 4 degrees eccentricity on the horizontal meridian and each contained a briefly presented Gabor patch. The Gabors were presented within a temporal Gaussian envelope with a standard deviation of 50 ms. This makes its effective duration on the order of 50 to 100 ms. After the first display (1000 ms), there was a delay with only the fixation cross (1000 ms); this was followed by a second display containing two noise patches (1000 ms). These displays also contained a briefly presented Gabor patch. After a brief delay with the fixation cross alone (250 ms), a 100% valid postcue appeared until the observer responded whether the orientation of either cued Gabor had changed from the first display to the second display. For Experiment 1, the postcue was identical to the precue. Only one response was required. Responses were given on a rating scale (likely-no, guess-no, guess-yes, and likely-yes) to measure an receiver operating characteristic (ROC) curve. Auditory feedback was provided for incorrect responses (180 Hz).

Each block consisted of 24 trials from one of the three conditions: set size 2, set size 1 left, and set size 1 right. A single experimental session included four set size 2 blocks, two set size 1 left blocks, and two set size 1 right blocks. Each observer performed practice sessions in which the Gabor contrast was lowered gradually until performance was stable around 80% correct for set size 1. For all experiments, the contrast used individual subjects ranged from 24% to 35% and the mean contrast for each experiment ranged tightly between 29% to 30%. Observers then completed 10 sessions at this near-threshold contrast, resulting in 1920 trials overall per observer. Each session took 20 to 25 minutes, and typically two sessions were run back-to-back within an hour. We also collected four sessions with high-contrast Gabors (80% contrast) from each observer to assess performance with highly visible stimuli. Inadvertently, in Experiment 2, one observer did not complete two low-contrast sessions and another observer did not complete two high-contrast sessions. In addition, in Experiment 4, one observer did not complete three high-contrast sessions.

**Noise Movies**. The “movies” had *1/f* noise in space and time and played for 1000 ms with an effective frame rate of 30 Hz. The movies were generated as follows: each frame was first populated with independent Gaussian noise at each pixel, with zero mean and unit variance. The frame was then filtered using a 2D Fourier transform such that the amplitude of each spatial frequency component *f_s_* was proportional to *1/f_s_*. Then, the whole movie was similarly filtered in time so that the amplitude of each temporal frequency *f_t_* was proportional to *1/f_t_*. The pixel values were then rescaled to have a standard deviation of 0.12 (a relatively low luminance contrast). The local contrast of each frame was attenuated at the edges by a linear ramp down to zero beginning 0.5 degrees from the nearest edge. Before the experiment, 2000 different noise movies were generated and were randomly drawn from for each trial.

**Gabors**. The Gabor patches had spatial frequency of 1 cycle/degrees and were windowed by a 2D Gaussian with a standard deviation of 0.5 degrees and truncated to a total width of 2 degrees. The Gabor could appear anywhere within the noise image, as long as the edges of the truncated width were at least 0.5 degrees from the edges of the noise. The Gabor's contrast was modulated in time by a Gaussian envelope with a standard deviation 50 ms. Thus, the effective duration was 50 to 100 ms. The time of maximal contrast was chosen from a uniform distribution, excluding the first and last 200 ms of the movie, but constrained to appear at the same time on both sides of the stimulus display to avoid the possible advantage of an attention switching strategy. Orientations were drawn uniformly from two sets of nonoverlapping standards (11.25 degrees, 56.25 degrees, 101.25 degrees, and 146.25 degrees) and (33.75 degrees, 78.75 degrees, 123.75 degrees, and 168.75 degrees). These standards were offset so that the same orientation was never present on both sides at once. The set of values used for each side varied randomly so that no orientation was associated with a side. Importantly, the orientation on one side was independent of the orientation on the other side.

**Apparatus**. The stimuli were displayed on a calibrated, flat-screen CRT monitor (19 inch ViewSonic PF790). This display was viewed from a distance of 60 cm, had a resolution of 832 × 624 pixels, and was refreshed at a rate of 120 Hz. The display had a peak luminance of 104 cd/m^2^, a black level of 3.9 cd/m^2^ due to room illumination, and the white had an CIE xy-chromaticity of (0.33 and 0.36). The display was controlled by a Mac Mini with system 10.6.8, using Psychophysical toolbox version 3.0.11 (Brainard, 1997), and MATLAB version 2012a (MathWorks, Natick, MA, USA).

**Eye position**. Fixation was required during the stimulus displays. On all trials, eye position was recorded using an Eyelink II, 2.11 with 250 Hz sampling (SR Research, Ontario, Canada). The position of the right eye was recorded for all trials, and trials were included for analysis only if fixation was confirmed. When fixation failed, observers were alerted with five consecutive high frequency tones and the trial was aborted. The percentage of aborted trials for each observer in each experiment ranged from 1.7% to 14% with an overall mean including all experiments of 5.7 ± 0.8%.

### Analysis

Observers responded with one of four key presses that indicated likely-no, guess-no, guess-yes, or likely-yes. These ratings were used to form an ROC function and performance was summarized as the percent area, *A’*, under the ROC function. *A’* is equivalent to the percent correct measured by forced-choice paradigms (Green & Swets, 1966). To estimate *A’*, the trapezoid method was used to avoid making distributional assumptions (Macmillan & Creelman, 2004) and was converted to a percentage. The difference in *A’* between set sizes 2 and 1 is our primary measure of the effect of divided attention. We refer to this as the relevant set-size effect. The statistical analysis focused on whether the set-size effect differed from zero in each experiment (a one sample *t*-test) and whether it differed from experiment to experiment (a two sample *t*-test with unequal variance). All statistical comparisons were two-tailed to make them consistent with the reported 95% confidence intervals (CIs).

## Experiment 1: Basic change detection

Our first experiment was designed to estimate the magnitude of the set-size effect in a version of change detection that is typical of the literature (e.g. Keshvari, van den Berg, & Ma, 2013). We used a precue to manipulate relevant set size rather than vary display set size. There were two stimulus displays separated by a blank. In each display, there was a stimulus on each side of fixation. For set size 1, if a change occurred, it was restricted to the precued side; for set size 2, the change could occur on either side and the observer had to make a single decision for the whole display. Given that the task is made up of two possible events that can map to the same response (a many-to-one mapping) this is sometimes called a compound task (Sperling & Dosher, 1986) and is commonly used in visual search. No set-size effect is expected if all processing stages have unlimited capacity (perception, memory, and decision).

### Method

The method was as described in the General Method section. The specific task is shown in Figure 2. The first and second stimulus displays contained a briefly presented Gabor on both the left and the right side. On 50% of the trials, a change in orientation of the Gabor of 90 degrees occurred on one of the relevant sides. In the relevant set size 1 blocks, the change could occur on only the precued side, and the uncued side always remained unchanged in orientation. In relevant set size 2 blocks, the change could occur on either side but not on both. The observer's task was to make a yes-no response as to whether a change had occurred anywhere.

### Results

**Figure 3.**
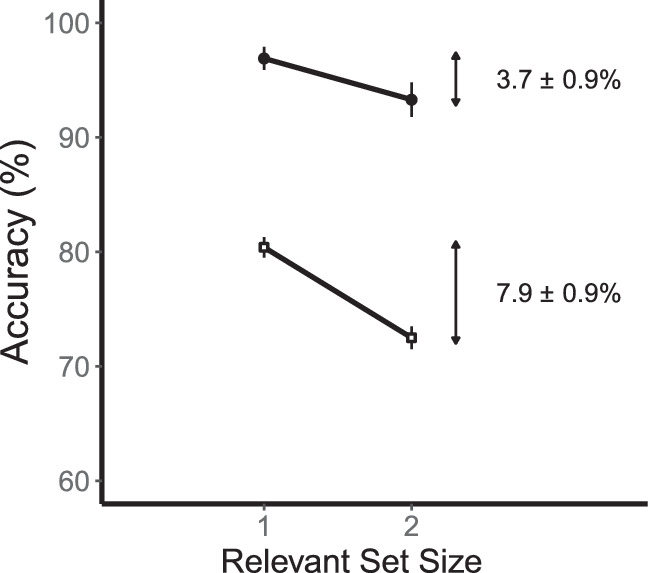
Results for basic change detection (Experiment 1). A graph of performance for relevant set sizes 1 and 2. Low-contrast conditions are shown by open squares and high-contrast conditions are shown by filled circles. There are reliable set-size effects for both contrast conditions. Error bars are the standard error of the mean.

The effects of relevant set size on accuracy (collapsed across sides) are shown in Figure 3. Consider first the low-contrast conditions shown by the open symbols. Performance was better for relevant set size 1 (80.4 ± 0.9%) than for relevant set size 2 (72.5 ± 1.0%). This is a reliable difference of 7.9 ± 0.9% (95% CI = 5.8, 10.0, *t*(11) = 8.36, *p* < 0.001). Now consider the high-contrast conditions shown by the filled symbols. Performance was better for set size 1 (96.9 ± 1.0%) than for set size 2 (93.3 ± 1.5%). This is a reliable difference of 3.7 ± 0.9% (95% CI = 1.6, 5.7, *t*(11) = 3.92, *p* = 0.002). For comparisons between experiments, we collapsed these two contrast conditions. The combined set-size effect was 5.8 ± 0.7 (95% CI = 4.3, 7.3, *t*(11) = 8.73, *p* < 0.001).

### Discussion

The results of the basic change detection experiment are consistent with similar studies in showing a set-size effect (Keshvari et al., 2013; Luck & Vogel, 1997; Scott-Brown & Orbach, 1998). In particular, the results are consistent with prior studies showing such an effect for set sizes 1 vs. 2 (Bae & Flombaum, 2013; Williams et al., 2013). Thus, even for two stimuli, one or more component processes must be limiting performance with multiple stimuli.

## Experiment 2: Postcues

In Experiment 2, we addressed the role of decision in change detection. Simple change detection as in Experiment 1 includes dependencies across sides because different events can lead to the same response (e.g. a change on the left or on the right will lead to a change response). This many-to-one mapping complicates the interpretation of the results because it obfuscates the source of information used in the decision (Braun & Julesz, 1998; Shaw, 1980; Sperling & Dosher, 1986). For example, two decisions might be required for set size 2 while only one decision is required for set-size 1. Therefore, in Experiment 2, each stimulus judgment was made an independent task (called a dual task or a concurrent task; Sperling & Dosher, 1986), and a postcue was used to sample one of these separate tasks. This results in a one-to-one mapping between stimulus and response (illustrated in the schematic in Figure 1B). On each side, a target can occur independently with 50% probability. This makes the stimulus displays for relevant set size 1 identical to relevant set size 2. The precue is the only difference between the set-size conditions. If the result of Experiment 1 is due to only the effect of the compounded decision error, then the set-size effect should be eliminated in Experiment 2.

### Method

**Figure 4.**
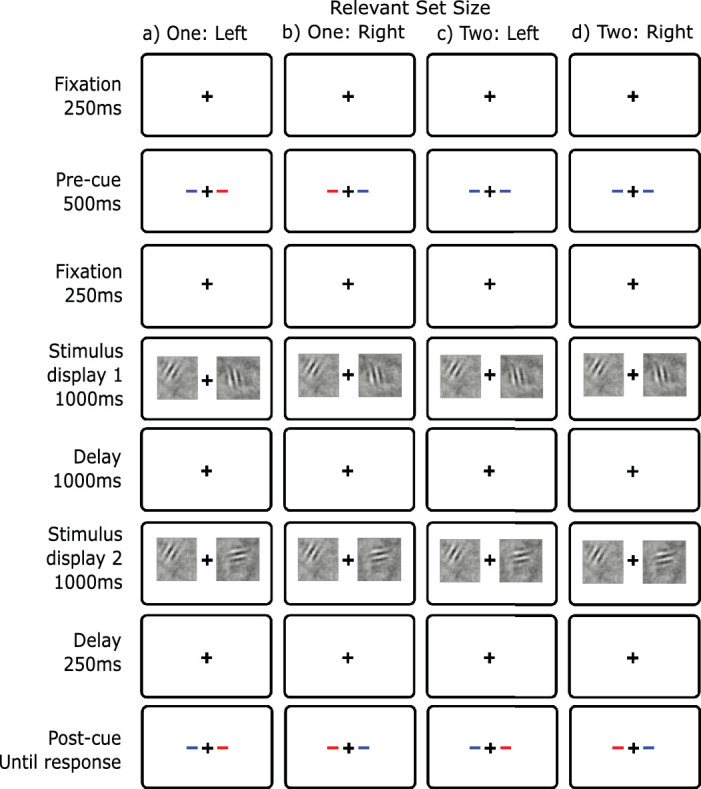
Display conditions for change detection with a dual-task procedure and postcue (Experiment 2). Blue is the cue color. (**A**) Relevant set size 1 - left: the left side was precued with 100% validity as the side that would be tested on this trial. (**B**) Relevant set size 1 - right: the right side was precued as relevant. (**C**) Relevant set size 2 - left: both sides were precued as potential response sides and the left side was later postcued for response. (**D**) Relevant set size 2 - right: both sides were precued as relevant and the right side was later postcued for response.

The General Method was used except that (a) the presence of a change was independent on the left and right side—changes occurred on one or both sides in both of the set-size conditions, and (b) observers had to respond to whether a change occurred within the postcued side only. The observer used two independent sets of response keys corresponding to the left and right side (but only one response was made on each trial). See Figure 4 for examples of this procedure. The postcue appeared 250 ms after the end of the second stimulus display (test). Because the brief Gabor patch could appear any time within the noise display, the mean stimulus onset asynchrony (SOA) between the test Gabor and the postcue was 750 ms with a range of 450 to 1050 ms. Such a relatively “late” postcue was used so that it did not help with retrieval, comparison, or decision about one side. Instead, the postcue indicates which of the decisions is relevant for this trial.

### Results

**Figure 5.**
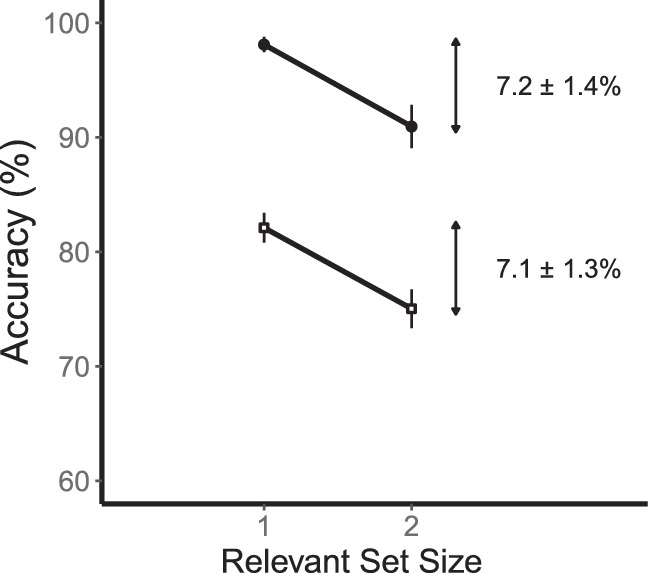
Results for change detection with a postcue (Experiment 2). Accuracy is shown for relevant set sizes 1 and 2. Low-contrast conditions are shown by open squares and high-contrast conditions are shown by filled circles. There are reliable effects for both contrast conditions.

The effect of relevant set size on accuracy is shown in Figure 5. For the low-contrast condition, performance was better for set size 1 (83.6 ± 1.2%) than for set size 2 (76.4 ± 1.8%). This is a reliable difference of 7.1 ± 1.4% (95% CI = 4.1, 10.1, *t*(11) = 5.21, *p* < 0.001). For the high-contrast condition, performance was also better for set size 1 (98.2 ± 0.6%) than for set size 2 (91.7 ± 1.9%). This is a reliable difference of 6.6 ± 1.5% (95% CI = 3.4, 9.8, *t*(11) = 4.50, *p* < 0.001). Collapsing across contrast conditions, the combined set-size effect was 6.9 ± 1.3% (95% CI = 4.0, 9.7, *t*(11) = 5.37, *p* < 0.001).

Our primary question in Experiment 2, was to determine whether the change in procedure (dual-task and postcue) to isolate a single decision reduced the set-size effect relative to Experiment 1. Collapsing across contrasts, the set-size effects were insignificantly larger in Experiment 2 (overall mean = 6.9%, 95% CI = 4.0, 9.7) than in Experiment 1 (overall mean = 5.8%, 95% CI = 4.3, 7.3). This is an unreliable difference (in the wrong direction) of −1.0%, *t*(17) = 0.73, *p* = 0.48. Thus, this change in procedure did not reliably change the set-size effect.

**Congruency**. The results of this experiment can be further analyzed by the congruency of the stimulus events at each location on each trial. Congruent trials have the same stimulus event (e.g. a change) occurring at both locations. Incongruent trials have different stimuli events occurring at each location (i.e. a change on one side and not on the other). Effects of congruency are evidence of interactive processing of the two stimuli (e.g. Navon & Miller, 1987) or selection errors between the stimuli (Yantis & Johnston, 1990). However, in this and the following experiments, there were relatively small or no congruency effects on performance (see Appendix). Thus, there is little sign of interactive processing or selection errors.

**Orientation similarity and perceptual grouping**. When two orientations are presented together, sometimes they can form a single perceptual representation or group (Silvis & Shapiro, 2014). If such perceptual grouping were to occur for our observers, it would undermine our assumption of testing one versus two stimuli. Due to our stimulus design, there were never identical orientations on both sides in a given stimulus display. However, there are still pairings that might be grouped into either corners or almost parallel lines. Despite this possibility, there was no evidence of perceptual grouping (see Appendix).

### Discussion

In this study, the dual task and postcue did not reduce set-size effects relative to Experiment 1. This lack of effect is similar to some previous studies (e.g. Luck & Vogel, 1997; Wheeler & Treisman, 2002) although others have found that set-size effects are reduced by a similar change in procedure (e.g. Beck & van Lamsweerde, 2011; Hollingworth, 2003). This literature is examined more closely in the General Discussion. In sum for this task, decision appears to not be a major limit on the set-size effect.

## Experiment 3: Retention-interval cue

In Experiment 3, the goal was to determine whether effects before memory retrieval contribute to the set-size effect. This experiment was identical to Experiment 2 with the postcue, except that an additional cue—labeled a retention-interval (also called a retro cue) — was added between the two stimulus displays (e.g. Griffin & Nobre, 2003). The retention-interval cue matched the postcue in indicating the relevant stimulus. This task therefore required observers to retrieve from memory only the Gabor orientation on the relevant side, and to make a single comparison decision on the relevant side before responding. This concept is illustrated in the processing schematic in Figure 1C. In short, this cue should eliminate any effect of capacity limits in retrieval or comparison.

Observers still had to perceive and encode the two stimuli from the first display, and they had to store these two stimuli in memory until the retention-interval cue. Thus, Experiment 3 has the same perception, memory encoding, and initial memory storage requirements as Experiment 2, but different memory retrieval and comparison demands. If perception, memory encoding, or memory storage of the two orientations is the limiting factor causing the set-size effect, then that effect should persist with a retention-interval cue. If, however, memory retrieval or comparison is the limiting factor, then the set-size effect should be eliminated.

There are two versions of the storage hypothesis that make different predictions than a limit based simply on storage capacity. The first we call selective maintenance. By this hypothesis, there are maintenance processes, such as rehearsal, that operate during the retention interval. The retention-interval cue allows for selective maintenance of the relevant stimulus for the portion of the retention interval following the cue. Another way to think of this hypothesis is as a kind of directed forgetting (see MacLeod, 1998, for a review), where the retention-interval cue designates the relevant stimulus as to-be-remembered and allows the irrelevant stimulus to be forgotten (or actively removed from storage), thereby reducing what must be retained (Souza, Rerko, & Oberauer, 2014). Thus, this hypothesis predicts that the retention-interval cue should reduce the set-size effect.

The second hypothesis we call selective transfer. Consider the multiple-store model presented by Sligte, Scholte, and Lamme (2008). They proposed that the relevant visual memory consists of both a high capacity, fragile store (Fragile VSTM), and a lower capacity, durable store (Traditional VSTM). Fragile VSTM is held to be durable enough to last at most a few seconds. Consequently, the retention-interval cue could allow the selective transfer of information about the relevant stimulus into the more durable Traditional VSTM store. Thus, this view also predicts that the retention-interval cue should reduce the set-size effect.

In summary, different storage hypotheses make different predictions about the influence of the retention-interval cue on the set-size effect. The storage capacity hypothesis predicts no influence of the cue on the set-size effect. In contrast, the selective maintenance and selective transfer hypotheses predict a reduced set-size effect.

### Method

**Figure 6.**
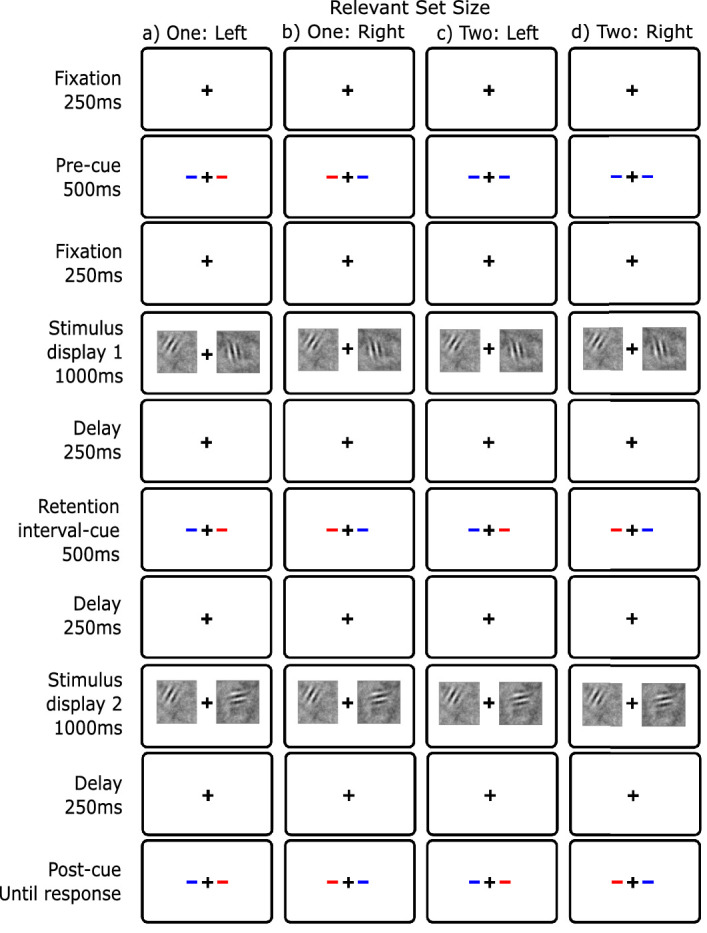
Display conditions of change detection with a retention-interval cue (Experiment 3). Blue is the cue color. The retention-interval cue appeared between the two displays and matched the postcue. (**A**) Relevant set size 1 - left, (**B**) relevant set size 1 - right, (**C**) relevant set size 2 - left, (**D**) relevant set size 2 - right.

The stimuli in this experiment were identical to those in Experiment 2. The only procedural change was the additional retention-interval cue, which was always identical to the postcue. For relevant set size 1, this new cue provided no additional information. For relevant set size 2, the observer knew which side was relevant during the retention interval and thereafter. Examples of the procedure are shown in Figure 6.

### Results

**Figure 7.**
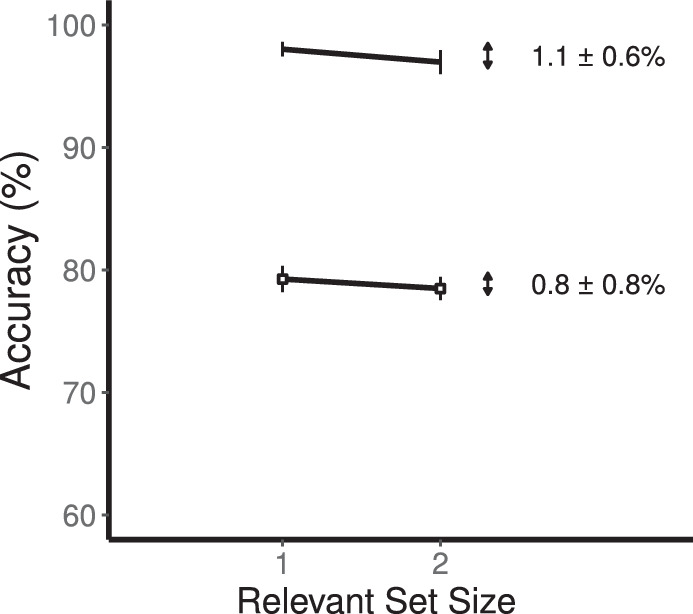
Results for change detection with a retention-interval cue (Experiment 3). Graph of accuracy as a function of relevant set size. Low-contrast conditions are shown by open squares and high-contrast conditions are shown by filled circles. These set-size effects are smaller than found for Experiment 2.

The effect of set size is shown in Figure 7. For the low-contrast condition, there was no reliable difference between set size 1 (79.5 ± 1.0%) and set size 2 (78.7 ± 0.9%). This is a difference of 0.9 ± 0.7% (95% CI = −0.7, 2.4, *t*(11) = 1.26, *p* = 0.23). For the high-contrast condition, there was also no reliable difference between set size 1 (98.0 ± 0.6%) and set size 2 (97.0 ± 1.0%). This is a difference of 1.0 ± 0.6% (95% CI = −0.2, 2.2, *t*(11) = 1.81, *p* = 0.10). Collapsing across contrast conditions, the combined set-size effect was a marginally reliable 0.9 ± 0.5% (95% CI = −0.1, 2.0, *t*(11) = 2.04, *p* = 0.066).

Our primary question in Experiment 3, was to determine whether the introduction of a retention interval cue reduced the set-size effect relative to Experiment 2. In fact, the set-size effect in Experiment 3 was reliably smaller than found in Experiment 2. Collapsing across contrast conditions, the set-size effect in Experiment 3 was 0.9% (95% CI = -0.01, 2.0) and was smaller than the set-size effect in Experiment 2 of 6.9% (95% CI = 4.0, 9.7). This reduction in the set-size effect is a reliable 5.9% (*t*(14) = 4.34, *p* < 0.001) and represents the bulk of the 6.9% effect found in Experiment 2.

### Discussion

The little or no set-size effects in Experiment 3 suggest that there is relatively little capacity limit up to and including the initial storage of the stimuli. In the extreme, for two simple stimuli, there is no limit in perception nor for memory encoding or initial storage. Instead, the limiting process must be primarily one of the later storage processes (selective maintenance or selective transfer), retrieval, or comparison.

## Experiment 4: Local recognition

**Figure 8.**
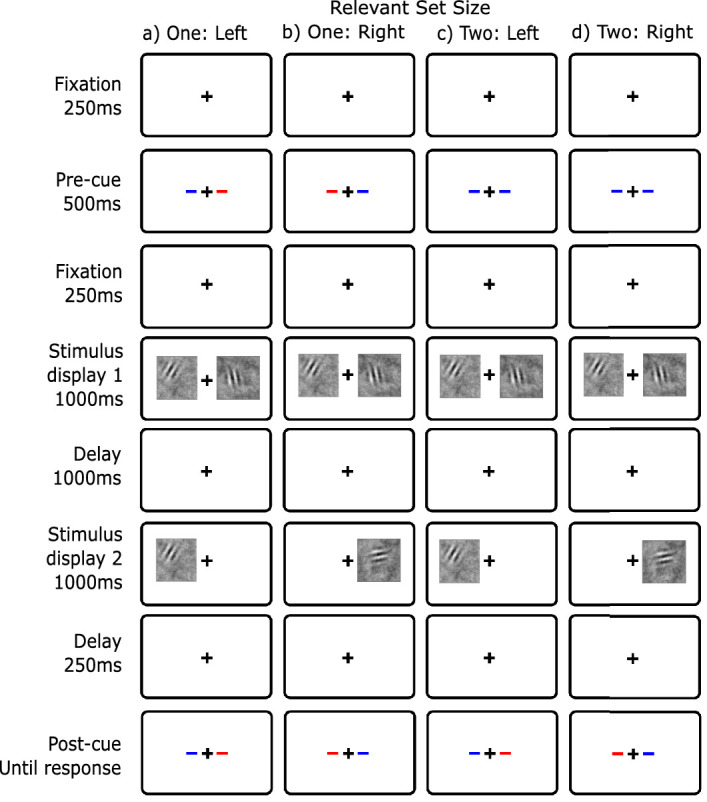
Display conditions for change detection with local recognition (Experiment 4). Blue as the cue color. (**A**) Relevant set size 1 - left, (**B**) relevant set size 1 - right, (**C**) relevant set size 2 - left, (**D**) relevant set size 2 - right.

In Experiment 4, we used a local recognition task (also called single-probe recognition): the first stimuli are presented on both sides followed by a test display with only one stimulus presented on the response side (Figure 8). The task is called local recognition to distinguish it from global recognition (e.g. Oberauer, 2003). In local recognition, the task is to compare the probe to a specific stimulus. Whereas in global recognition, the task is to compare the probe with all of the stimuli in the display. Local recognition is similar to Experiment 3 in that the observer must make only one memory retrieval and comparison. The new feature of this experiment is that now hypotheses, such as selective maintenance and selective transfer, no longer predict a reduction in the set-size effect. The information indicating the relevant stimulus comes after the retention interval as part of the test display. If this local recognition task eliminates the set-size effect, it would be consistent with capacity limits in either retrieval or comparison. This paradigm is similar to experiments where a cue is presented simultaneously with the second (or test) stimulus display (Luck & Vogel, 1997; Makovski, Sussman, & Jiang, 2008; Wheeler & Treisman, 2002).

### Method

The stimulus displays were identical to Experiment 2 except that the second stimulus display contained only one stimulus on the relevant side (see Figure 8).

### Results

**Figure 9.**
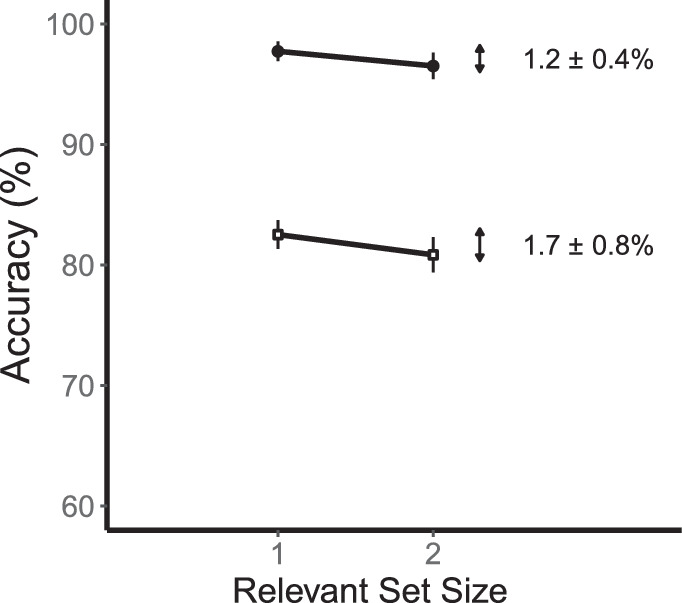
Results for change detection with local recognition (Experiment 4). A graph of accuracy as a function of relevant set size. Low-contrast conditions are shown by open squares and high-contrast conditions are shown by filled circles. These set-size effects are smaller than found for Experiment 2.

The effect of relevant set size is shown in Figure 9. For the low-contrast condition, there was a small but marginally reliable difference between set size 1 (82.6 ± 1.2%) and set size 2 (80.8 ± 1.5%). This is a difference of 1.7 ± 0.8% (95% CI = 0.0, 3.5, *t*(11) = 2.19, *p* = 0.051). For the high-contrast condition, there was also a small but reliable difference between set size 1 (97.7 ± 0.8%) and set size 2 (96.5 ± 1.1%). This is a difference of 1.2 ± 0.4% (95% CI = 0.3, 2.0, t(11) = 3.04, *p* < 0.011). Collapsing across contrast conditions, the combined set-size effect was a reliable 1.5 ± 0.4% (95% CI = 0.6, 2.3, *t*(11) = 3.65, *p* = 0.004).

Our primary question in Experiment 4, was to determine whether the use of a local recognition task reduced the set-size effect relative to Experiment 2. In fact, the set-size effect in Experiment 4 was reliably smaller than found in Experiment 2. Collapsing across contrast conditions, the set-size effect in Experiment 4 was 1.5% (95% CI = 0.6, 2.3) and was smaller than the set-size effect in Experiment 2 of 6.9% (95% CI = 4.0, 9.7). This reduction in the set-size effect is a reliable 5.4% (*t*(13) = 4.03, *p* = 0.001) and represents the bulk of the 6.9% effect found in Experiment 2.

### Discussion

In Experiment 4, local recognition, in which the second display included only one of the stimuli, showed a smaller set-size effect than did typical change detection, in which the second display included both of the stimuli rather than just one. The previous literature on local recognition is mixed. Wheeler and Treisman (2002) compared change detection with whole displays and with the single displays of local recognition. They found better performance with local recognition but it is not clear whether set-size effects were reduced. In contrast, Jiang, Olson, and Chun (2000) compared these conditions using larger set sizes and found worse performance with local recognition. They attributed this effect to the loss of configural information with the single display in local recognition. The current study minimizes the role of configural information, which may explain why our results were more like those found by Wheeler and Treisman.

The results of Experiment 4 are consistent with the results of Experiment 3 in showing that when only one retrieval and comparison must be made there is a diminished set-size effect. An alternative explanation for Experiment 3 is that the retention-interval cue between the stimulus displays changed the storage processing in some way. For example, the cue might have allowed either selective removal of the irrelevant stimulus information or selective transfer of the relevant stimulus information to a more durable memory. Finding similar results for Experiment 4, which did not have the retention-interval cue**,** rules out an explanation based solely on a difference in storage processes (Williams & Woodman, 2012; Zhang & Luck, 2008). Instead, the only hypotheses consistent with all experiments involve capacity limits in retrieval and/or comparison.

## General discussion

In this study of change detection, our goal was to find the primary locus of capacity limits with just two stimuli. Comparing one versus two relevant stimuli reveals the initial limits on processing relative to a single stimulus. In addition, we manipulated relevant set size to measure purely attentional effects, and measured both hard-to-discriminate and easy-to-discriminate stimuli to address experiments typical, respectively, of perception and memory.

### Summary of results

**Figure 10.**
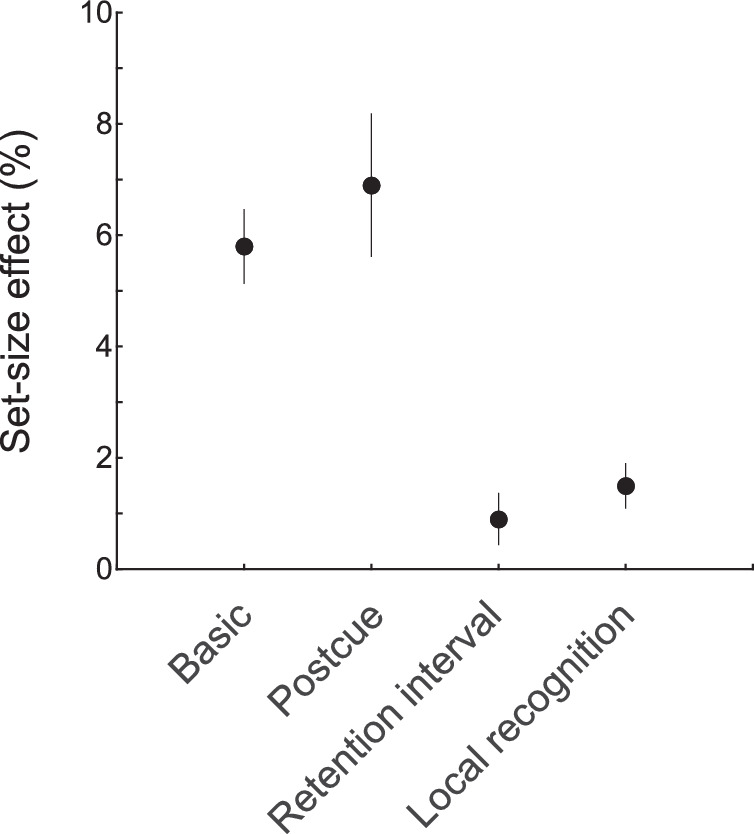
Magnitude of the effect of relevant set size for each of the four experiments. This figure combines the similar results for the low and high contrast conditions. Set-size effects are found for the basic and postcued experiments but a reduced effect is found for the experiments using a retention-interval cue or local recognition.

We measured effects of relevant set size on coarse orientation discrimination in four kinds of change detection as summarized in Figure 10. This figure combines the similar results obtained for low contrast and high contrast stimuli. For basic and postcued change detection (Experiments 1 and 2), there were set-size effects of 5.8% and 6.9% (an overall average of 6.3%): performance was worse for two relevant stimuli compared to one. These two tasks required the processing of two stimuli throughout perception, memory, and comparison so the effect could be due to any of these processing stages.

For change detection using a retention-interval cue (Experiment 3), there was a reduced set-size effect compared to Experiments 1 and 2. The observed effect was 0.9% compared to a mean effect of 6.3% in Experiments 1 and 2. For this task, both stimuli must be processed by perception, memory encoding, and satisfy the initial storage limits. But the cue allows only a single relevant stimulus to be processed by memory storage processes (e.g. memory maintenance), memory retrieval, and comparison. Thus, the reduction in set-size effect is consistent with a loci in one or more of the later processes.

For change detection using local recognition (Experiment 4), there was also a reduced set-size effect compared to Experiments 1 and 2. The observed effect was 1.5% compared to a mean effect of 6.3% in Experiments 1 and 2. For this task, two relevant stimuli must be processed by perception, memory encoding, and all aspects of memory storage. But only a single relevant stimulus must be processed by memory retrieval and comparison. Thus, this reduction of set-size effects is consistent with a loci in retrieval and/or comparison processes. To summarize our results into one comparison, for Experiments 1 and 2 that required multiple retrievals and comparisons, the mean set-size effect was 6.3%; and, for Experiments 3 and 4 that minimized retrieval and comparison, the mean set-size effect was reduced to 1.2%. Put another way, four fifths of the set-size effect were eliminated in Experiments 3 and 4.

### Generality of results thus far

There is obviously more to do to establish the generality of these results, some of which is discussed below. Nonetheless, the current studies establish the generality of how set-size effects depend on the task in two ways. First, they measured two memory tasks that required multiple retrievals and comparisons: the basic change detection task that required search for change, and the postcue paradigm that used a dual-task procedure to independently measure memory for each stimulus. Both tasks yielded relatively large set-size effects of around 6%. These tasks were compared to two other memory tasks that minimized retrieval and comparison: a task with retention interval cues and a task with local recognition. Both of these paradigms yielded relatively small set-size effects of around 1.2%. Thus, our results generalize to two tasks that maximize retrieval/comparison and to two tasks that minimize retrieval/comparison.

Second, for all four of our experiments, we have measured set-size effects for two stimulus conditions: (a) the low-contrast conditions which limited performance using low visibility stimuli typical of perception experiments; and (b) the high-contrast conditions that had clearly visible stimuli with accuracies of around 98% correct in relevant set size 1. Such highly visible stimuli are typical of memory experiments. Despite these differences in visibility and performance, there was a similar pattern of set-size effects: relatively large set-size effects for the conditions that required multiple retrievals and comparisons (Experiments 1 and 2), and relatively small set-size effects for the conditions that minimized retrieval and comparison (Experiments 3 and 4). Thus, our results for multiple experiments generalize across two quite different stimulus conditions.

**Figure 11.**
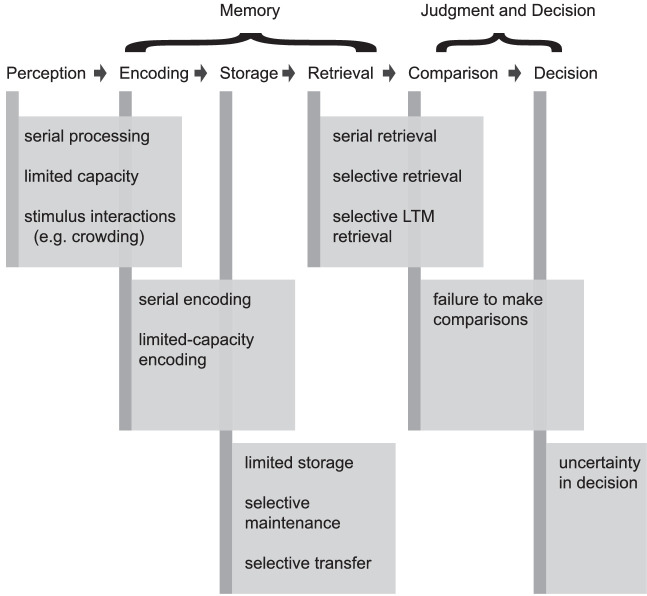
Summary of the main hypotheses for set-size effects for change detection that are considered in this article. The six processing stages from the introduction are shown at the top, and text boxes with hypotheses for each stage are shown below. The results are consistent with the hypotheses under retrieval and comparison.

In the next part of the discussion, we consider how these results relate to hypotheses specifying the processing locus that causes the set-size effect. The possibilities considered are summarized in Figure 11. At the top of the figure are the processing stages introduced in the introduction: perception, encoding, storage, retrieval, comparison, and decision. In text boxes below each processing locus, there is a list of the primary hypothesis considered in this article. For example, under perception, the hypotheses are serial processing, limited-capacity parallel processing, or stimulus interactions such as crowding.

### Implications for perception

By perceptual processing, we mean the immediate processing of the stimulus rather than any delayed processing that is based on memory. It is always challenging to separate effects of perception from the early effects of memory encoding and storage. In the experiments presented here, it was unnecessary to pursue this distinction owing to the inference of unlimited capacity across all of these early processing stages.

Many change detection experiments vary the number of stimuli in the initial stimulus display and therefore change the sensory input from condition to condition which can introduce unintended stimulus interactions, such as crowding (e.g. Parkes, Lund, Angelucci, Solomon, & Mortan, 2001). We avoided this potential confound by always presenting the same stimuli in all conditions. Such constant displays rule out non-attentional accounts, such as crowding.

Finding little or no effect of relevant set size for perception of simple features (e.g. luminance contrast or orientation) is consistent with results from detection or detection-like tasks (Bonnel, Stein, & Bertucci, 1992; White, Runeson, Palmer, Ernst, & Boynton, 2017). These results differ from the predictions of theories that posit a limited capacity in perception for divided attention tasks. At one extreme, are theories suggesting a serial process (or “bottleneck”) that allows only one stimulus to be identified at a time (Broadbent, 1958), whereas other theories are less severe suggesting a limited capacity or resource that is divided among relevant stimuli (e.g. Kahneman, 1973). These theories predict set-size effects because the stimuli are in competition for the limited processing capacity in perceptual stages. One explanation for these contrasting findings of limited and unlimited capacity is a two-stage theory that predicts unlimited capacity for processing in the first stage, and limited capacity for processing in the second stage (e.g. Hoffman, 1979; Scharff et al., 2011b; Treisman & Gelade, 1996).

In summary, our results are inconsistent with a capacity limit in the perception of simple features. If the stimuli were not both perceived, then the experiments using a retention-interval cue or local recognition could not have improved performance and reduced the set-size effect. Thus, for the case of feature processing and 1 vs. 2 stimuli, we can reject theories with limited-capacity perception (Kahneman, 1973; Pestilli et al., 2011). Of course, this does not rule out the possibility that such theories play a role with larger set sizes or for more complex stimuli.

### Implications for perception of the test stimuli

There are two issues concerning the perception of the test stimuli in the second display. First, one might propose that limited capacity processing for the test stimulus contributes to the set-size effects. This might particularly be the case because our experiments did not use long duration test displays. But there is good evidence against capacity limits for perception in this task. It is based on the little or no set-size effects in Experiments 3 and 4 that still included the perception of one versus two stimuli in the first display. If there are no effects on perception of the first display, it seems likely there would be no effects on perception of the second display.

The second issue is that the perception of the test display might interfere with memory of the first display, a possibility suggested by Makovski and Jiang (2007). Recent studies have made a case that such interference does contribute to set-size effects and to the reduction of those set-size effects with the retention-interval cue (e.g. Souza, Rerko, & Oberauer, 2016). The idea that selective attention can protect against visual interference is similar to ideas on object substitution masking (Enns & Di Lollo, 1997).

In our study, there is no sign of the test display interfering with memory of the first display. Such interference would be expected to confer an advantage on the condition with the retention-interval cue (Experiment 3) compared to local recognition (Experiment 4), but there was a similar lack of set-size effects for these two experiments. We suggest that such an interference effect was absent in our study because our displays included dynamic visual noise for an extended time before and after both stimuli. This kind of filled interval is likely to cause its own visual interference (Makovski, Shim, & Jiang, 2006) and thus precluded additional interference from the test display. Consequently, whereas interference from the test display probably can occur in change detection, it played little role in the current study.

### Implications for memory

In the general memory literature, the use of attentional cues to specify relevant and irrelevant stimuli (relevant set size) is similar to directed forgetting (for a review, see MacLeod, 1998). In this paradigm, participants are postcued to remember some items and to forget other items because these forget items will not be tested. In fact, though, all items are tested and forget items are poorly remembered compared to remembered items. Most of this work has been for verbal stimuli, but see Williams, Hong, Kang, Carlisle, and Woodman (2013) and Hourihan, Ozubko, and MacLeod (2009) for studies using visual stimuli. In this literature, there are three primary hypotheses to account for this kind of attentional effect in memory: selective rehearsal (Basden, Basden, & Gargano, 1993), retrieval inhibition (Bjork, 1989), or selective search in retrieval (Epstein, Massaro, & Wilder, 1972). Consider next specific memory hypotheses for the current study.

**Memory encoding.** Serial processing or limited-capacity processing at encoding means that insufficient stimulus information can be moved to storage. Sensory representations are retained only briefly after the stimulus event (e.g. Enns & Di Lollo, 1997; Palmer, 1988; Sperling, 1960) and therefore are unlikely to be encoded much beyond the first stimulus display in our task. The reduction of set-size effects by the retention-interval cue and by local recognition suggests that, in fact, the stimulus representations are successfully encoded into memory storage because they are available for later access. This is consistent with those who have found insensitivity to stimulus duration (Cowan, Elliott, Saults, Morey, Mattox, Hismjatullina, & Conway, 2005; Luck & Vogel, 1997; Sperling, 1960), or have otherwise argued against capacity limits in encoding (Rideaux et al., 2015; Rideaux & Edwards, 2016). This result is not compatible with encoding that is serial or has limited capacity (e.g. Becker et al., 2013).

In summary, our results are inconsistent with a capacity limit in memory encoding. If the stimuli were not both encoded, then the retention-interval cue (Experiment 3) and local recognition (Experiment 4) could not have improved performance and reduced the set-size effect. Thus, for a set size of 1 vs. 2, we can reject theories with limited-capacity encoding (Becker et al., 2013; Palmer, 1990). Of course, this does not rule out the possibility that these theories play a role with larger set sizes or different encoding conditions.

**Memory storage.** There are several ways that memory storage might mediate set-size effects. The simplest is a limit on storage capacity itself. Such a limit might be in terms of the number of stimulus representations (e.g. Zhang & Luck, 2008) or in terms of the quality of the representations (e.g. Keshvari et al., 2013). These hypotheses are inconsistent with the results of the experiments with a retention-interval cue or local recognition that reduce set-size effects despite identical storage requirements.

Two other ways that memory storage might mediate the set-size effect we have called selective maintenance and selective transfer. In selective maintenance, memory is improved by a limited-capacity maintenance process (e.g. rehearsal) that is applied to the representations of the relevant stimuli (e.g. Basden et al., 1993). In selective transfer, memory is improved by a limited-capacity process that transfers information about the relevant stimulus to a more durable memory storage (e.g. Sligte et al., 2008). Both of these hypotheses predict that retention-interval cues reduce set-size effects.

In a particularly relevant study by Williams et al. (2013), one versus two colored squares were presented and the precision of recall was measured for a single color. Performance for a set size of two colors was worse than for one color, but was improved when a retention-interval cue indicated that only one color was relevant. The authors argued that their results were evidence for a limited-capacity, selective maintenance process, such as selective rehearsal.

The problem with selective maintenance and selective transfer is that neither predicts that set-size effects are reduced by using a local recognition task (Experiment 4). Thus, these hypotheses are inconsistent with our results. One possible reason for the lack of effects for selective transfer is that we used a largely noise-filled retention interval that might have eliminated contributions from less durable memory stores.

**Memory retrieval.** Limited capacity in retrieval means that although both stimuli were encoded and stored adequately, the representations from the first stimulus display are not recovered from memory with sufficient accuracy for successful comparison (Shiffrin, 1970). Our results are consistent with such a limit in retrieval. The reduction of the effect of relevant set size by a retention-interval cue (Experiment 3) or by local recognition (Experiment 4) allows one to make a single memory retrieval rather than two. If multiple retrievals interfere with one another in some way, this can account for both the set-size effect and its reduction with an appropriate cue.

Several hypotheses for limited-capacity retrieval have been suggested in the literature. By the serial retrieval hypothesis (also called the retrival bottleneck hypothesis, Carrier & Pashler, 1995; Oberauer, 2018), only one retrieval is possible at a time. For a brief test display, this can result in retrieval failure for the second stimulus. A related idea is the retrieval head start hypothesis (Souza et al., 2016). By this hypothesis, a retention-interval cue can provide a head start for the retrieval process. This hypothesis has also been supported by the finding that delaying the response after a retention interval cue improves performance. Consider next the selective retrieval hypothesis (Epstein et al., 1972). By this hypothesis, selected context cues provide additional guidance for the retrieval of the relevant memory. One interesting variation on this idea is that the more specific context protects the retrieval process from interference from the other stimuli (Oberauer & Lin, 2016). Another variation of selective retrieval is the retrieval inhibition hypothesis (Bjork, 1989). This hypothesis focuses on selectively improving retrieval by inhibiting the memory of irrelevant stimuli. Finally consider the long-term-memory retrieval hypothesis (Beck, Peterson, & Angelone, 2007; Beck & van Lamsweerde, 2011). By this hypothesis, explicit retrieval cues, such as the retention-interval cue or the single test stimulus in local recognition, improve retrieval from long-term memory that supplements retrieval based on working memory alone.

These retrieval accounts clash with accounts of working memory that assert no retrieval for the stimulus representations held in the focus of attention (e.g. Cowan, 1988). By these accounts, memories for the stimuli in the focus of attention can be directly accessed for comparison to the stimuli in the second display. McElree (1998) has argued that this focus of attention is limited to as little as one object, whereas Cowan (1988) has argued that it can encompass several objects. In more recent reviews, Cowan (2011) has defended his proposal but has been criticized by Oberauer (2013) and the debate continues (e.g. Vergauwe & Langerock, 2017). If direct access to multiple objects is found to hold, then retrieval cannot be the limit for the results found here.

### Implications for comparison

The results with the retention-interval cue and with local recognition are also consistent with a limit in the comparison process. Only one comparison has to be made for these conditions. This hypothesis is supported by results showing that despite failing to detect a change, a subsequent probe about stimulus identity demonstrates that sufficient information was available in memory (Angelone et al., 2003; Farell, 1985; Fernandez-Duque & Thornton, 2000; Hyun et al., 2009; Mitroff et al., 2004; Simons et al., 2002). For example, Mitroff et al. (2004) showed that, despite encoding sufficient information about all of the relevant stimuli for a 2AFC task asking whether stimuli had been present in either display, observers still failed to detect changes. Findings such as this have been interpreted as showing that, when there are multiple comparisons to be made, one can retrieve the relevant memory but fail to make the correct comparison.

### Implications for decision

Decision can contribute to set-size effects when there is uncertainty in mapping stimuli to a specific response. As the number of relevant stimuli increases, additional uncertainty from each stimulus is included in the decision which consequently limits performance (Palmer et al., 2000; Sperling & Dosher, 1986; Swets, Tanner, & Birdsall, 1961). Change detection tasks are often structured such that all stimuli contain relevant information that must be integrated to make the decision. In Experiment 1, for example, observers were asked to detect a change occurring anywhere in the array, so that all locations are informative to the decision. In Experiment 2, by contrast, the postcue directed observers to the single stimulus relevant to their task.

In fact, the addition of a postcue and the independent tasks did not reduce set-size effects. Similar results have been found in previous change detection experiments with colored squares (Luck & Vogel, 1997; Wheeler & Treisman, 2002) and with letters (Becker, Pashler, & Anstis, 2000). In contrast to those results, there is evidence of a postcue effect in a study of Gabor patches (Wright, Green, & Baker, 2000). Therefore, the results with simple stimuli are unclear.

A somewhat different pattern of results has been found for two studies using familiar objects. Postcues reduced the set-size effects in an experiment using an array of familiar objects (Beck & van Lamsweerde, 2011) and in an experiment using familiar objects in natural scenes (Hollingworth, 2003). These experiments also used relatively long study display durations to encourage the use of long-term memory. In addition, Beck and van Lamsweerde provided specific evidence for the role of long-term memory and argued that the postcue effects are due to encouraging retrieval from long-term memory.

There is an alternative view of the comparison process worth mentioning. In Experiment 2, a correct response requires accurate information about the location of the change as well as whether a change occurred. If location information was imperfect, one would expect a decline in performance in Experiment 2 compared to Experiment 1. That was not found. The analysis of congruency effects is relevant to this possibility. If location information is unreliable, then one would expect better performance on congruent trials than on incongruent trials. For congruent trials, both locations require the same response so unreliable information about location does not affect performance. That is not true for incongruent trials. In fact, there were no congruency effects in any of our experiments (see Appendix). This is consistent with location information not limiting performance in these experiments. This is perhaps not surprising because the differences in location were maximized (left versus the right side of fixation) and the use of relevant set size minimized changes in context.

Why might there be no effect of set size on decision in the current experiments? One possibility is that these memory tasks depend on discrete representations rather than the continuous representations as assumed by typical signal detection models. A simple two-state representation does not necessarily predict an uncertainty effect on decision (see the high threshold theories described in Palmer et al., 2000). This idea is supported by a recent study of color change detection task by Rouder et al. (2008; see also related studies of long-term memory, Province & Rouder, 2012). Unfortunately, the evidence for this possibility is not universal. Ricker, Thiele, Swagman, and Rouder (2017) used similar methods on an orientation-based change detection task and found no evidence for a discrete representation. Thus, the discrete representation model appeared to be a viable account of the results found here for a decision, but there is doubt that this model generalizes to all change detection tasks.

### Generalization to larger set sizes

How do the current results and interpretations generalize to larger set sizes? To address this, it helps to compare our study to Experiment 1A of Souza, Rerko, & Oberauer (2014). It had many of the same goals and methods as our study along with a critical difference. Their goal was to distinguish alternative hypotheses for the effect of retention interval cues. The hypotheses addressed several possible loci, including storage and retrieval/comparison/decision. Souza and colleagues also considered ideas about interference. Like our Experiment 3, they focused on how set-size effects were modulated by the retention interval cues. The key difference is that instead of a baseline of basic change detection, they used a baseline of local recognition. This important change minimizes any contribution from hypotheses involving retrieval, comparison, or decision.

Here is a brief description of their relevant results. For set size 2, performance with or without retention interval cues matched performance with set size 1. This absence of a retention-interval cue effect is similar to our results showing similar performance with retention interval cues and local recognition. For set size 4, there was a set-size effect for local recognition of about 10%. This effect was reduced with retention interval cues to about 4%. This difference between the combination of retention interval cues and local recognition, and local recognition alone was the critical result of their experiment. For set size 6, the pattern of results was similar: a set-size effect for local recognition of about 18%, which was reduced with retention interval cues to about 10%. In the original paper, they argued that these results supported the hypothesis that retention interval cues allowed the irrelevant memory trace to be removed from storage. In two follow-up articles (Shepherdson, Oberauer, & Souza, 2018; Souza, Rerko & Oberauer, 2016), they described two other possibilities: protection from visual interference and a retrieval head start.

The critical question for comparison to our study is what the set-size effect would have been for a basic change detection task instead of local recognition. We expect such a set-size effect would have been quite a bit larger than they obtained for local recognition. That is what we obtained for set size 2. If it is also obtained for larger sets sizes, that would support a general role of retrieval, comparison, or decision processes in the set-size effect. In summary, our study and Souza et al. (2014) have the same results for the overlapping conditions. We extended their results by making comparisons to the basic change detection task. Their results extend ours by exploring larger set sizes.

### Generalization to display set size

Our experiments are somewhat unusual in manipulating relevant set size instead of display set size. What would we expect for similar experiments that manipulate display set size? The strongest result would be similar magnitude set-size effects for manipulations of relevant set size and display set size. Such a match would be consistent with both little effect of stimulus interactions in display set size, and little effect of imperfect cueing in relevant set size. Previously, we have found such a match for set-size effects in accuracy visual search (Palmer, 1994), on set-size effects with response time visual search (Palmer, 1998), with a comparison of simultaneous and sequential conditions (Scharff, Palmer, & Moore, 2011a), with a comparison of single-task and dual-task conditions with object recognition (Popovkina, Palmer, Moore, & Boynton, 2021); and with a comparison of single-task and dual-task conditions with Gabor patch detection (Palmer, White, Moore & Boynton, 2020). In all of these cases, we have shown for widely separated stimuli and 100% valid cues well before the display, that there are similar set-size effects for relevant set size and display set size. Because the current experiments were similar to the Gabor detection experiments of Palmer et al. (2020), we argue it is likely that the current results would generalize to display set size.

But suppose this case is different and the effects found with relevant set size do not generalize to display set size. One explanation is that relevant set size might underestimate display set-size effects because the cues are not fully effective. But that possibility does not seem likely because our cueing procedure (highly visible endogenous cues presented well before the display) has been effective in the studies cited above. Another explanation for not having similar results with display and relevant set size is because increasing display set size causes additional stimulus interactions. Such interactions have been found for large set sizes possibly due to crowding (Palmer, 1994; Zelinsky, 1999). It is that possibility that motivated our use of relevant set size. Under those larger set-size conditions, relevant set size provides the more accurate estimate of purely attentional set-size effects.

### Generalization to fine orientations

In the main conditions of this study, we measured set-size effects under a variety of cueing conditions using a coarse orientation judgment limited by low contrast and noise. Would the pattern of results be similar for judgments that were limited instead by fine orientation changes? While those experiments remain to be done, there has been a number of studies examining memory for orientation using the “psychometric function” approach of visual psychophysics (e.g. Bays & Husain, 2008; Keshvari, et al., 2013; Palmer, 1990). In these studies, accuracy is measured as a function of the orientation difference (some with differences as large as 90 degrees). For example, Salmela and Sarrinen (2013) measured change detection with orientation differences that varied from 5 degrees to 30 degrees. They were able to describe the results as a simple function of the change in orientation multiplied by (1/set size). In a second experiment using the delayed estimation procedure (also called continuous report), they found the variability of the report also varied inversely with set size. Recently, Lilburn, Smith, and Sewell (2019) found similar results and provided a detailed discussion of possible theoretical interpretations. Thus, set-size effects are consistent for a range of orientations and procedures. A common theory for coarse and fine orientations seems likely.

The second issue in this generalization is how orientation discrimination is affected by external noise. There are situations in which noise increases the magnitude of spatial cueing effects (Smith, 2000). Although there are few relevant memory experiments (but see Santana, Godoy, Ferreira, & Galera, 2013), Baldassi and Burr (2000) investigated the effect of external noise on a visual search task with orientation judgments of Gabor patches. Specifically, in their “identification” task, they presented from 2 to 16 Gabor patches with all vertical distractors and a single target tilted to the left or right. Observers had to indicate whether the target was tilted left or right. For both no external noise and a range of noise levels, they found that set-size effects varied by the same (1/set size) factor. External noise did cause a decline in performance, but the set-size effect remained proportionally the same. Thus, external noise in this study limited performance but did not change the set-size effect. In summary, there is some evidence that fine orientation and coarse orientation in noise have similar set-size effects. This makes it plausible that the results we found for coarse orientation in noise generalize to fine orientation.

### Generalization to color

In this article, we focus entirely on the case of orientation, but all of our interpretation has assumed that orientation is not a special feature. Many studies of change detection use salient and highly discriminable colors and thus color would be a natural generalization. The question of whether color is different from orientation has been raised in the literature. In a series of papers asking whether encoding into visual short-term memory occurs in series or in parallel, Becker and colleagues found different results for color and orientation (Becker et al., 2013; Liu & Becker, 2013; Mance, Becker, & Liu, 2012; Miller, Becker, & Liu, 2014). In a local recognition task, they compared performance for stimuli presented simultaneously versus sequentially. If encoding has unlimited capacity, then there should be no difference between these conditions, but if encoding has limited capacity, then performance should be better in the sequential condition. They found that for color – but not orientation – the performance was equivalent between the simultaneous and sequential conditions. Instead, orientation had a sequential advantage with better performance for the sequential condition compared to the simultaneous condition. They suggested that color, but not orientation, can be encoded in parallel for one versus two stimuli.

What to make of these results? First, they indicate that orientation and color are sometimes processed differently. One possible explanation is that there were stimulus interactions in the orientation experiments and not the color experiments. This is possible because these experiments all manipulated display set size rather than relevant set size. Second, the sequential advantage found for orientation appears inconsistent with our data. In our local recognition task (Experiment 4), there was nearly unlimited capacity for orientation judgments of two stimuli. Again, the use of relevant set size in our experiments might be the critical difference. Moreover, the differences between color and orientation found by Becker and colleagues are in the wrong direction to predict a different pattern of results for color in our task. They found even less capacity limitation for color and our surprising result is the little capacity limitation for orientation. In sum, we think it likely that the results found here for orientation generalize to color.

### Working hypothesis

We close with a working hypothesis about the sources of set-size effects in change detection in brief displays. It is based on the results of our experiment, the results of Souza, et al. (2014) and what is known about perceptual crowding (e.g. Whitney & Levi, 2011). For set size 2 versus 1, performance is primarily limited by retrieval and comparison. With an increase to set sizes 4 and 6, there are additional limits due to storage and/or interference that build on the limits of retrieval and comparison. Finally, beginning by set size 8 (if not earlier), performance is also limited by perceptual crowding, which becomes increasingly important at yet larger set sizes. Although this picture is probably incomplete, we propose that these are three of the most important sources of set-size effects in change detection.

## Conclusion

We investigated set-size effects in change detection for coarse orientation. Our goal was to find the primary locus of the initial capacity limits revealed by set sizes 1 and 2. Relevant set size was used rather than display set size to measure purely attentional effects and minimize other phenomena, such as crowding. In Experiment 1 with basic change detection, there was an effect of relevant set size: Performance was worse with two relevant stimuli compared to a single relevant stimulus. This effect was also found for Experiment 2 using change detection with a dual-task procedure and a postcue. But the results were different for Experiment 3 with a 100% valid retention-interval cue between the stimulus displays, and for Experiment 4 that used local recognition to test memory for a single stimulus. For these two experiments, the set-size effect was much reduced relative to the first two experiments. From this pattern of results, the capacity limit with just two stimuli must be largely due to memory retrieval and/or comparison. For these set sizes, our experiments rule out perception, memory encoding, and memory storage as the locus for the bulk of the capacity limits. This result for 1 vs. 2 stimuli is inconsistent with the predictions of a variety of theories including limited-capacity perception (e.g. Kahneman, 1973; Pestilli et al., 2011) and limited-capacity memory storage (e.g. Keshvari et al., 2013; Zhang & Luck, 2008).

## Appendix: Further analyses of all experiments

### Stimulus timing

For all experiments other than experiment 3, the second stimulus and postcue occurred beyond the time limits of iconic memory (Sperling, 1960). In experiment 3, however, the retention-interval cue appeared 250 ms after the end of the stimulus display. The Gabor could be present up to the last 200 ms of the stimulus display, resulting in the possibility that in some trials the retention-interval cue could appear within 450 ms. We suspect that this is beyond the limits of iconic memory for our displays, but what if it is not? It would then be possible for the observer to encode only one stimulus from the first display and lead to improved performance in the relevant set size 2 condition relative to other experiments.

To test the possibility that stimulus timing affected performance, we correlated the timing of the Gabor target within the first stimulus display with the performance on that trial. If the presence of the target in iconic memory at the time of the retention-interval cue was of benefit, then performance should improve the later the Gabor is presented within the interval (positive correlation). None of the observers showed a reliable correlation in experiment 3 or any other experiment (mean correlation coefficient = 0.01, *p* > 0.1 with Bonferroni correction). Thus, there is no evidence of an effect of when the retention-interval cue appeared relative to the stimuli.

### Congruency

The results of our dual-task experiments (2, 3, and 4) can be further broken down by congruency of the stimuli events at each location on each trial. Congruent trials have the same stimulus event (e.g. a change) at both locations; incongruent trials have different stimulus events at each location (e.g. a change on one side and not on the other). The presence or absence of congruency effects indicates the degree of independence across locations. If performance is independent, then there should be no difference in performance between congruent and incongruent trials. On the other hand, if there is a trial-by-trial dependency across sides, then there should be a difference in performance between congruent and incongruent trials (e.g. Bonnel, Stein, & Bertucci, 1992). Such an effect is indicative of a divided attention effect beyond an accuracy difference across set-size conditions. Examples of congruency effects are described in Navon and Miller (1987).

The congruency effects for our three dual-task experiments (experiments 2–4) are given in Table 1 with separate rows for each experiment and for each contrast condition, and separate columns for set sizes 1 and 2. Consider first experiment 2. Recall, it was the only one of these three experiments that had substantial set-size effects. It was also the only experiment that showed congruency effects. Moreover, these effects were specific to set size 2 that had both stimuli relevant. The congruency effects in units of percent area under the ROC were 2.1 ± 0.7% and 3.7 ± 1.1% for low and high contrast respectively. In contrast, the set size 1 congruency effects were near zero. For the other two experiments, there were no reliable congruency effects in any condition. Given the use of a retention interval cue or local recognition, this is to be expected if the source of these effects is in retrieval, decision, or response.

**Table 1.** Congruency effects in experiments 2, 3, and 4.

Experiment	Contrast	Set size 1	Set size 2
2	Low	−0.1 ± 0.8%	2.1 ± 0.7%*
2	High	−0.2 ± 0.6%	3.7 ± 1.1%*
3	Low	0.7 ± 0.8%	−0.5 ± 0.9%
3	High	−0.1 ± 0.5%	1.1 ± 0.7%
4	Low	−0.5 ± 0.5%	−0.3 ± 0.7%
4	High	0.7 ± 0.8%	−0.5 ± 0.7%

Congruency effects in experiments 2, 3, and 4 are broken down by contrast condition. These congruency effects were the difference between congruent and incongruent conditions in units of the percent area under the ROC. As marked by an asterisk, the only reliable effects were in experiment 2 for set size 2.

This pattern of congruency effects being different under conditions of divided attention (relevant set size 2) versus conditions of selective attention (relevant set size 1) has been reported before (Logan & Gordon, 2001; Palmer, White, Moore & Boynton, 2020). It is consistent with divided attention allowing some interaction between two relevant stimuli that selective attention can prevent when one of the stimuli is relevant and the other is irrelevant. Moreover, this interpretation suggests that congruency effects contribute to the set-size effects observed here because they reduce performance in set size 2 but not in set size 1. But at most, they contribute half of the effect observed in experiment 2. In summary, there were congruency effects in only set size 2 and only when both stimuli were relevant at the time of retrieval, decision, and response.

### Orientation similarity and perceptual grouping

Several findings suggest that perceptual grouping did not play a significant role in the present experiments. First, performance was no better or worse in local recognition than with the retention-interval cue. Removing the reference of the second side stimulus in local recognition should have removed any benefits of perceptual grouping because the context has changed. This predicts a smaller set-size effect in retention-interval cued change detection. However, the set-size effect was similar in these two experiments suggesting that perceptual grouping was not improving performance for the set size 2 conditions with the retention-interval cues relative to local recognition.

Second, if certain sets of orientations were more conducive to grouping, then there should be differences between these pairs. There were two possible differences between angles on the two sides (22.5 degrees and 67.5 degrees) and the mean performance was the same in each case (22.5 degrees: mean = 86.1, SE = 0.4; 67.5 degrees: mean = 86.1, SE = 0.4). A 3-way ANOVA 4 (experiment) × 2 (contrast) × 2 (orientation distance) found main effects of experiment and contrast but not of orientation difference, F(1, 179) = 0.71, *p* = 0.40.

Third, when the side of the change is not relevant, as in experiment 1, perceptual grouping might improve performance if any stage of perception, memory, or comparison was limited because it is only the one grouped set of stimuli that must be encoded, stored, and retrieved for comparison. Any change in this stimulus from the first to the second display would be reported as a change. By making the locations independent in experiment 2, this strategy was less helpful. The magnitude of the set-size effect was similar in experiments 1 and 2 suggesting that perceptual grouping was not helpful as a strategy for reducing the effective number of stimuli.

Why are there no effects of perceptual grouping? Any potential benefit of perceptual grouping might have been eliminated due to the presence of noise or to the randomized location of the stimuli from first to second stimulus display.
